# Efficiency of Desiccation, Biomass Production, and Nutrient Accumulation in Zuri and Quênia Guinea Grasses in Integrated Crop–Livestock Systems and Second-Crop Maize

**DOI:** 10.3390/plants13223250

**Published:** 2024-11-20

**Authors:** Bruno de Souza Marques, Kátia Aparecida de Pinho Costa, Hemython Luís Bandeira do Nascimento, Ubirajara Oliveira Bilego, Eduardo Hara, Rose Luiza Moraes Tavares, Juliana Silva Rodrigues Cabral, Luciana Maria da Silva, José Carlos Bento, Breno Furquim de Morais, Adriano Carvalho Costa, Tiago do Prado Paim

**Affiliations:** 1 Department of Animal Sciences, Federal Institute of Goiano, Rio Verde 75900-000, GO, Brazil; bruno.marques@estudante.ifgoiano.edu.br (B.d.S.M.); breno.furquim@estudante.ifgoiano.edu.br (B.F.d.M.); adriano.costa@ifgoiano.edu.br (A.C.C.); tiago.paim@ifgoiano.edu.br (T.d.P.P.); 2 Department of Agricultural Sciences/Agronomy, Federal Institute of Goiano, Rio Verde 75900-000, GO, Brazil; luy.mari@hotmail.com (L.M.d.S.); jose.bento@ifgoiano.edu.br (J.C.B.); 3 Centro Tecnológico Comigo (CTC), Rio Verde 75900-000, GO, Brazil; hemythonluis@comigo.com.br (H.L.B.d.N.); ubirajarabilego@comigo.com.br (U.O.B.); eduardohara@comigo.com.br (E.H.); 4 Department of Plant Production, University of Rio Verde, Rio Verde 75900-000, GO, Brazil; roseluiza@unirv.edu.br (R.L.M.T.); juliana.cabral@unirv.edu.br (J.S.R.C.)

**Keywords:** soil cover, *Panicum maximum* cv. BRS Quênia, *Panicum maximum* cv. BRS Zuri, sustainability, *Zea mays*

## Abstract

Modern agriculture faces the challenge of increasing production without expanding cultivated areas, promoting sustainable practices that ensure food security and environmental preservation. Integrated crop–livestock systems (ICLSs) stand out as an effective strategy, diversifying and intensifying agricultural production in a sustainable manner, ensuring adequate soil cover, and improving nutrient cycling efficiency. Thus, this study aimed to explore and compare integrated crop–livestock systems with Zuri guinea grass (*Panicum maximum* cv. BRS Zuri) and Quênia guinea grass (*Panicum maximum* cv. BRS Quênia) against the conventional soybean/maize succession method in a tropical region, and how these systems affect biomass decomposition, C:N ratio, nutrient cycling, and fertilizer equivalents. A field experiment was conducted in two phases: the first in the second-crop season and the second in the main season, using a randomized block design with four replicates. The treatments consisted of two ICLS systems, one with Zuri and Quênia guinea grasses established after soybean, and a succession system with maize established after soybean. The results indicated that Quênia guinea grass showed greater desiccation efficiency, with an injury rate of 86.5% at 21 days, 8.5% higher compared to Zuri guinea grass. In terms of biomass, Zuri and Quênia guinea grasses had average productions of 7021.1 kg ha^−1^, which was 43.25% higher compared to maize biomass. The biomass decomposition of the grasses was faster due to their lower C:N ratio, resulting in greater nutrient release into the soil. Both forage grasses (Zuri and Quênia guinea grasses) are suitable for integrated crop–livestock systems, as they showed similar biomass production and nutrient accumulation. Soybean yield was not influenced by the different cropping systems, showing similar results between the biomass of Zuri and Quênia guinea grasses and maize. However, grass biomass enriches the soil more through the return of fertilizer equivalents, which in future studies could be considered for the reduction of mineral fertilizers, ensuring greater sustainability of agricultural systems.

## 1. Introduction

The growing demand for food, driven by the global population increase, requires a transformation of agricultural practices to make them more sustainable. Agriculture faces the challenge of increasing production without expanding cultivated areas, minimizing environmental degradation, and promoting food security [1]. Sustainable intensification becomes essential to balancing agricultural production and the conservation of natural resources while maintaining economic viability for farmers [2].

In this context, integrated crop–livestock systems (ICLSs) emerge as an effective strategy, promoting the sustainable diversification and intensification of agricultural production in the same area [3]. These systems combine agricultural crops and pastures in rotation or intercropping, providing a range of ecological and economic benefits [4]. The integration of forage species with grain crops, such as soybean, in no-till systems leverages the synergy between soil, plants, and animals, resulting in higher productivity, better land use, and sustainability [5].

Among the forage species used, those of the *Panicum maximum* genus, such as Zuri guinea grass (*Panicum maximum* cv. BRS Zuri) and Quênia guinea grass (*Panicum maximum* cv. BRS Quênia), have stood out due to their favorable characteristics. For livestock, they present great potential and high animal performance, even during the off-season [6], when precipitation and temperatures are not favorable for forage production. For agriculture, these forages play a crucial role in maintaining the sustainability of production, due to their biomass production for the no-till system [7], better weed control [8], nutrient accumulation and release in the soil for subsequent crops [7,9,10], improvements in soil health [5,11], increased and diversified production [12], and contributions to reducing greenhouse gas emissions [3]. These forages also have great potential to increase soil carbon (C) and nitrogen (N) stocks [13,14], in addition to promoting greater water and soil conservation [15].

Additionally, animals grazing on tropical forages contributes to the resilience and sustainability of agricultural systems [16], as animals convert forage into nutrients for their development, and part of these nutrients returns to the system through residues (feces and urine), which contribute to nutrient recycling and the addition of organic matter to the soil, promoting the maintenance of soil fertility [17].

On the other hand, second-crop maize cultivation is a common practice in Central Brazil. However, the cover biomass produced by maize has been considered insufficient for good soil cover and nutrient cycling compared to tropical forages [18]. Replacing maize with forages in the off-season can significantly improve nutrient cycling and subsequent soybean productivity, contributing to a more sustainable and efficient agricultural system [9,19].

A desiccation efficiency is crucial to ensuring that the forage biomass fully dries and forms a suitable cover for subsequent crops, such as soybeans. Efficient desiccation facilitates effective nutrient cycling by accelerating the decomposition of cover crops and promoting nutrient release to the soil, benefiting the following planting season [18]. This process depends significantly on the forage cultivar’s sensitivity to herbicides, as some cultivars, like Quênia guinea grass, exhibit morphological traits that enhance their responsiveness to herbicides, ensuring complete desiccation and transition to a decomposable biomass state [10,20].

In this context, the choice of management practices in the off-season for soil cover biomass production in the no-till soybean system is essential to optimizing system efficiency, ensuring greater agricultural sustainability and productivity. Thus, the use of Zuri and Quênia guinea grasses in ICLSs can be an effective strategy to promote practices that ensure efficient food production, environmental preservation, and profitability for farmers.

Therefore, this study aimed to explore and compare integrated crop–livestock systems with Zuri and Quênia guinea grass with the conventional soybean/maize succession method in a tropical region, and how these systems affect biomass decomposition, C:N ratio, nutrient cycling, and fertilizer equivalents. The hypothesis is that Zuri and Quênia guinea grasses improve soil cover biomass production, nutrient accumulation, and fertilizer equivalents, which may affect soybean productivity compared to maize monoculture.

## 2. Results

**Figure 1:**
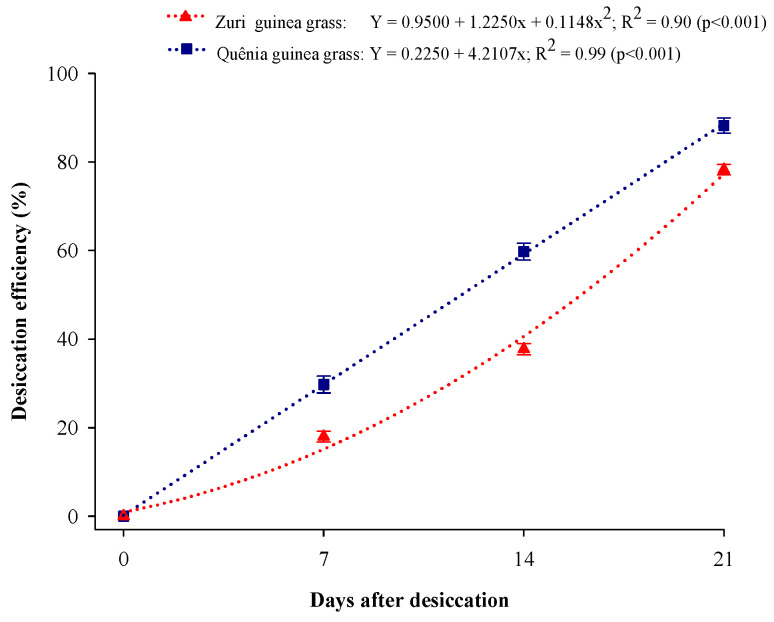
Desiccation efficiency of Zuri and Quênia guinea grass.

At 7, 14, and 21 days, the desiccation efficiency was 29.5%, 59.5%, and 86.5% for Quênia guinea grass and 18.0%, 31.5%, and 78.0% for Zuri guinea grass (Figure 1). At 21 days of desiccation, Quênia guinea grass showed an 8.5% increase in efficiency compared to Zuri guinea grass.

**Figure 2:**
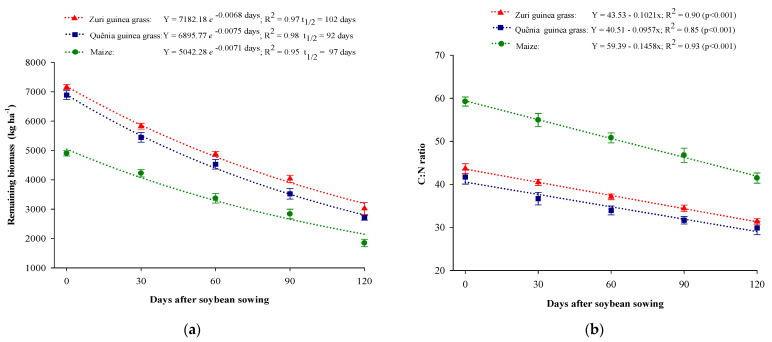
Remaining biomass (**a**) and C:N ratio (**b**) of maize and *Panicum maximum* cultivar cropping systems during soybean development (from 0 to 120 days).

The highest biomass production was obtained with Zuri and Quênia guinea grasses, with an average production of 7021.1 kg ha^−1^, which was 43.25% higher compared to maize biomass. The same pattern was observed for the remaining biomass (Figure 2a), where, at the end of the soybean development cycle (120 days), the remaining biomass was 1846.7 kg ha^−1^ for maize, 2709.4 kg ha^−1^ for Quênia guinea grass, and 3022.2 kg ha^−1^ for Zuri guinea grass. In terms of half-life, Quênia guinea grass and maize had a lower value (92 days) compared to Zuri guinea grass, which was 101 days.

The C:N ratio showed a linear decrease for all cropping systems with biomass decomposition times. For all evaluated periods, the highest C:N ratio was observed for maize, with an initial value of 59 and a final value of 41. Quênia guinea grass had the lowest ratio, with an initial value of 40 and a final value of 29 (Figure 2b).

The different soil cover biomasses influenced nutrient accumulation, with an exponential reduction during the soybean development cycle (120 days), as shown in Figure 2a. The highest accumulations of nitrogen, phosphorus, potassium, and sulfur were obtained in the soil cover biomass of Zuri and Quênia guinea grass, while the lowest accumulation was observed in maize biomass at all cultivation times, indicating a lower potential for nutrient accumulation.

**Figure 3:**
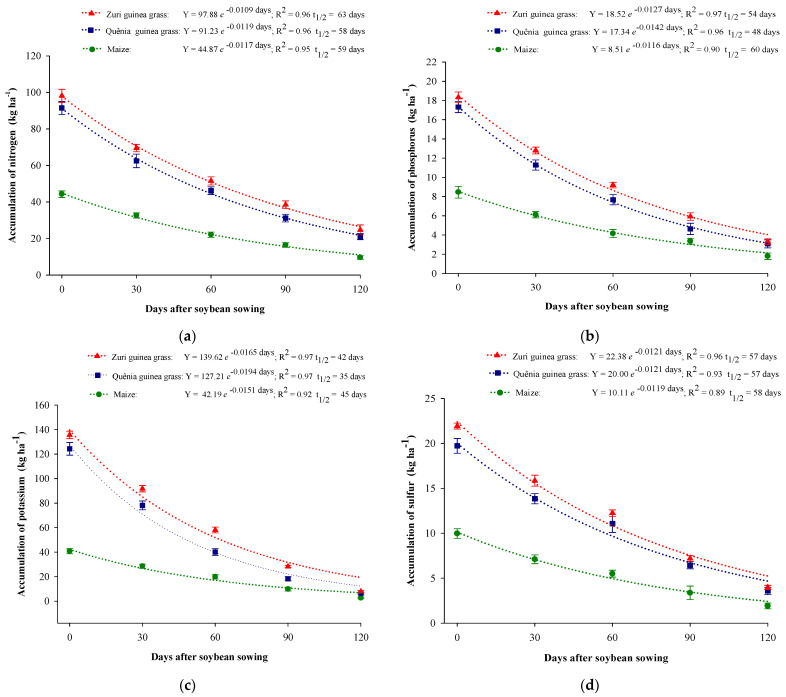
Accumulation of nitrogen (**a**), phosphorus (**b**), potassium (**c**), and sulfur (**d**) in the biomass of maize and *Panicum maximum* cultivar cropping systems during soybean development (from 0 to 120 days).

As biomass decomposed throughout the soybean development cycle, there was a percentage release of nitrogen of 79.70%, 77.42%, and 75.42%; of phosphorus of 79.11%, 82.39%, and 82.03%; of potassium of 94.36%, 94.92%, and 94.34%; and of sulfur of 80.04%, 82.55%, and 82.24% in the biomass of maize, Quênia, and Zuri guinea grasses, respectively (Figure 3).

Potassium had the shortest half-life (t½) compared to other nutrients, with 45 days for maize biomass, 42 days for Zuri guinea grass, and 35 days for Quênia guinea grass, indicating that this nutrient is rapidly released and in a high percentage, above 94%, in all biomasses [18]. The longest half-life (t½) for most nutrients, except nitrogen, was observed in maize biomass, indicating a low release rate in this crop residue. Following this, the systems with Zuri and Quênia guinea grass biomass showed shorter half-lives, with Quênia guinea grass presenting the shortest half-life (t½) for all nutrients (Figure 2).

**Figure 4:**
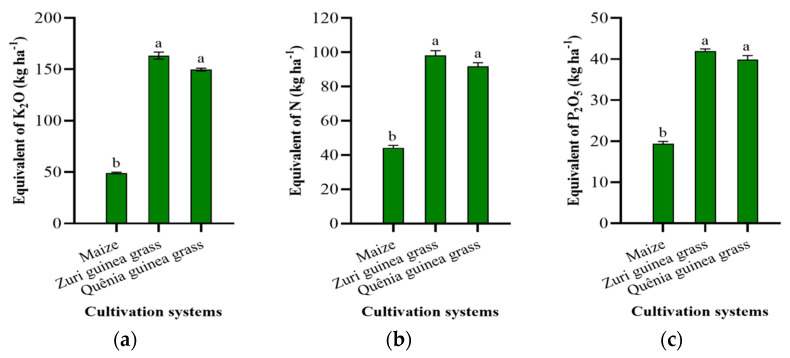
Equivalent contents of K_2_O (**a**), N (**b**), and P_2_O_5_ (**c**) in the biomass of maize, Zuri, and Quênia guinea grasses. Means followed by different letters differ significantly according to Tukey’s test at 5% probability.

In Figure 4, the equivalent contents of nitrogen (N), phosphorus (P_2_O_5_), and potassium (K_2_O) in the biomass of the different cropping systems are shown. Zuri and Quênia guinea grass stood out compared to maize, presenting the highest nutrient returns to the soil.

For nitrogen, Zuri and Quênia guinea grasses showed increases of 121.66% and 106.60%, respectively. For phosphorus (P_2_O_5_), the increase was 116.70% and 104.64%, respectively, and for potassium (K_2_O), there was a more pronounced increase of 232.15% and 204.47%, respectively, compared to the return of fertilizers in maize biomass.

The agronomic characteristics and soybean yield (Table 1) were not influenced by the different cropping systems, showing similar results between the biomass of Zuri and Quênia guinea grasses and maize.

**Figure 5:**
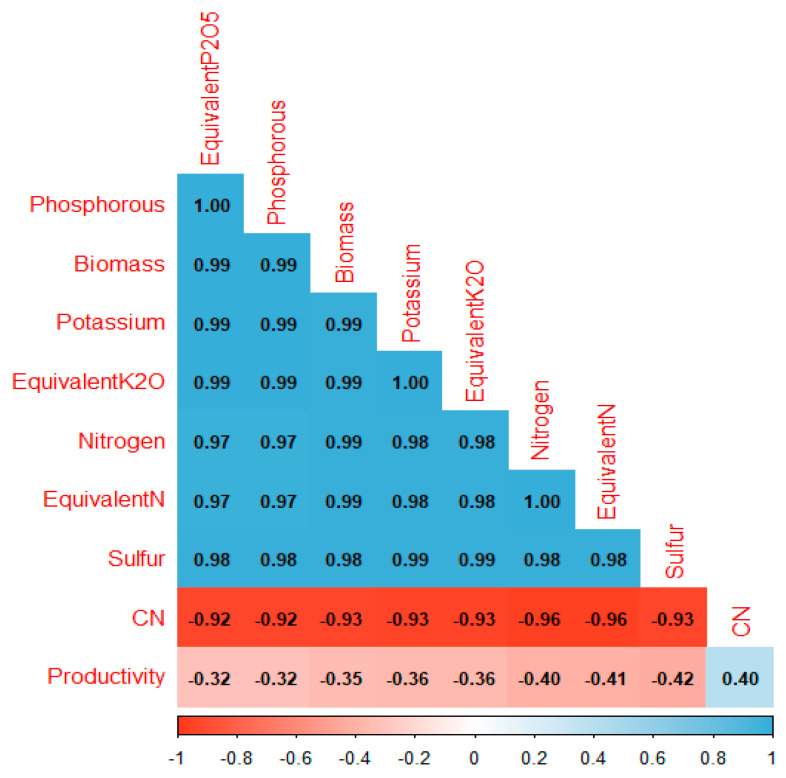
Pearson correlation (r) between parameters. Positive correlations are represented by blue backgrounds, and negative correlations are represented by red backgrounds. Parameters: phosphorous: phosphorus concentration, biomass: biomass accumulation, potassium: potassium concentration, equivalent K_2_O: equivalent concentration of K_2_O, nitrogen: nitrogen concentration, equivalent N: equivalent concentration of nitrogen, sulfur: sulfur concentration, CN: carbon/nitrogen ratio, productivity: crop yield, equivalent P_2_O_5_: equivalent concentration of P_2_O_5_.

Through correlation analysis (Figure 5), it was possible to identify the formation of three distinct groups of variables. The first group consisted of phosphorus, biomass, potassium, equivalent K_2_O, nitrogen, equivalent N, and sulfur, which showed high and positive correlations among themselves. The second group was formed by the C:N ratio (carbon/nitrogen ratio), which had high and negative correlations with the variables in group 1. The third group was represented by the yield variable, which showed moderate correlations with the other groups.

Through principal component analysis (PCA), we can observe (Table 2) that the first two components together explain a significant amount of the total data variation (98.15%).

**Figure 6:**
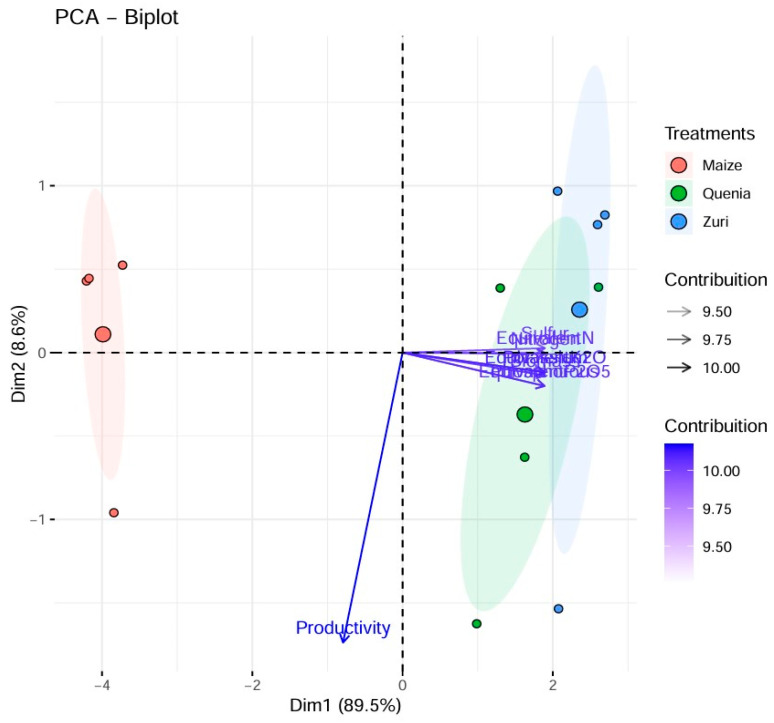
Two-dimensional scatter plot of principal component analysis and scores of the 10 variables, observations, and treatment means for initial biomass nutrient accumulation and soybean productivity. Maize: maize biomass; Quênia: Quênia guinea grass biomass; Zuri: Zuri guinea grass biomass; biomass; CN; nitrogen; phosphorus; potassium; sulfur; equivalent N; equivalent P_2_O_5_; equivalent K_2_O; productivity.

The first component (PC1) explained 89.53% of the total variation, showing high and positive correlations with the variables biomass, nitrogen, phosphorus, potassium, sulfur, equivalent N, equivalent P_2_O_5_, and equivalent K_2_O, and a high negative correlation with the C:N ratio, which can be graphically observed by the horizontal displacement of the arrows (Figure 6). The second component (PC2-vertical) explained 8.62% of the total variation, showing a significant negative correlation with the productivity variable (−0.91), as observed by the vertical displacement of the arrows.

Through principal component analysis, it was possible to observe that the Zuri and Quênia guinea grass treatments diverged from the maize treatment for the variables most related to the first principal component, with higher C:N values for maize and lower C:N values for the other variables associated with this treatment. Regarding the second component, little difference between treatments for productivity was observed graphically.

The number of components to be used to explain the behavior of the variables and the discrimination of treatments is based on the eigenvalue and the percentage of variance explained by the components. Based on the eigenvalue, components with values greater than 1 are considered. According to the criterion that takes into account the percentage of explanation, the number of components that jointly explain at least 70% of the total variation should be used. The first two components were presented, clearly discriminating the treatments in the first component. In the second component, there was little variation among treatments. These results are consistent with the univariate analysis but provide a global view of the experiment.

## 3. Discussion

The higher desiccation efficiency of Quênia guinea grass, reaching 86.5% at 21 days, is attributed to its morphology, characterized by an abundance of narrow to intermediate leaves with a width of 2 to 3 cm and lower tuft formation, making it more susceptible to glyphosate [8,21]. Similar results were observed by Silva et al. [20], who reported a desiccation efficiency of 91% for Quênia guinea grass in the same period. In contrast, Zuri guinea grass showed a desiccation efficiency of only 78% at 21 days, likely due to its morphology, characterized by taller growth and larger tuft formation, making it less susceptible to glyphosate [22].

The use of glyphosate in this study aligns with widely adopted agronomic desiccation practices, particularly in crop–livestock integration systems, where efficient soil cover and nutrient cycling are essential. Glyphosate was utilized due to its ability to promote uniform forage mortality and facilitate biomass decomposition, ensuring proper preparation for subsequent planting, as previously demonstrated by Silva et al. [23] and Jenkins et al. [24]. Silva et al. highlighted the role of glyphosate in the management of Panicum maximum cv. BRS Zuri, showcasing benefits such as reduced weed competition and improved nutrient cycling without compromising the performance of subsequent crops [24].

Although many producers resist using grasses such as those of the *Panicum* genus due to their tall growth and tufted habit, and the difficulty in management [19], the high desiccation efficiency observed, as reported by Silva et al. [20], demonstrates that these grasses can be a viable alternative. Moreover, the appropriate timing of desiccation and proper management can mitigate operational challenges during sowing, preventing issues such as soil compaction and interference with planting operations [12].

After desiccation, Zuri and Quênia guinea grasses produced 7182 and 6886 kg ha^−1^ of biomass, respectively, demonstrating their high production potential and ability to generate a substantial amount of biomass, similar to the results observed by Silva et al. [18]. Zuri guinea grass is characterized by its tall growth, tufted habit, and vigorous regrowth, resulting in significant dry matter production per hectare [25]. On the other hand, Quênia guinea grass has an intermediate growth habit that is smaller than Zuri guinea grass, but it has the advantage of producing more leaves and fewer stems, resulting in a high leaf-to-stem ratio [26]. Therefore, both forages showed great production potential, resulting in efficient and prolonged soil cover. This cover is crucial for maintaining soil thermal and water stability [27], benefiting the development of subsequent crops [18].

**Figure 7:**
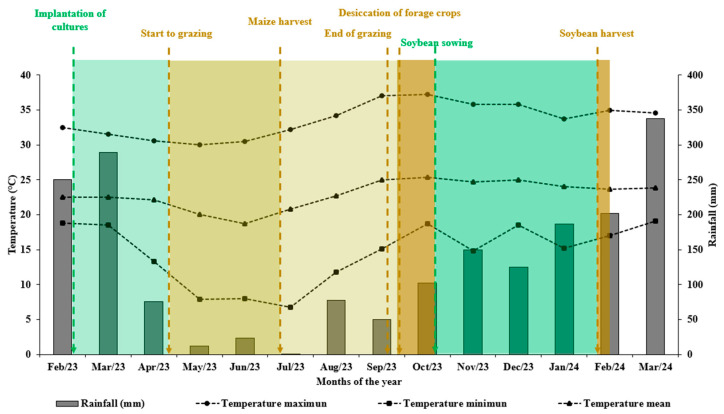
Monthly precipitation and temperature recorded from February 2023 to March 2024, at the Centro Tecnológico COMIGO in Rio Verde–GO, Brazil.

It is worth noting that favorable climatic conditions in August and September 2023, with an average precipitation of 64 mm (Figure 7), contributed to higher biomass production. Typically during this period, especially in August, there are conditions of little or no water availability, which hinders forage regrowth after animal grazing.

The lower biomass production of maize compared to forages is due to the smaller residue left on the soil after grain production. It is also noteworthy that maize cultivation does not provide good soil cover compared to tropical forages, potentially negatively affecting soil cover and making it more susceptible to erosion and moisture loss, especially under adverse climatic conditions [9,28]. The structure of maize, mainly consisting of stalks, results in less biomass on the soil after grain harvest [29].

The biomass produced by Zuri and Quênia guinea grass had C:N ratios of 43.53 and 40.51, respectively, which were lower compared to maize biomass (59.39), thus facilitating the decomposition process, making nutrient mineralization faster and more efficient, and ensuring persistent and effective soil cover throughout the soybean development cycle. The high leaf-to-stem ratio promotes more intense organic matter degradation, favoring nutrient mineralization [9]. C:N ratios between 12 and 25 are ideal for mineralization, while values above 50 contribute to immobilization [30].

The higher C:N ratio of maize can be explained by the large amount of recalcitrant material, such as stalks, cobs, and husks, left on the soil after grain harvest. These structures contain high concentrations of lignin, a fiber that confers resistance to decomposition by microorganisms [29]. Thus, maize biomass decomposes more slowly, prolonging nutrient availability in the soil but also delaying mineralization. Truong and Marschner [30] and Silva et al. [7] observed C:N ratios of maize above 50, contributing to nutrient immobilization and making them less available for subsequent crops.

The differences in biomass production and C:N ratios between the biomass produced by maize and by Zuri and Quênia guinea grasses have significant implications for the sustainability of agricultural systems. In regions such as the Brazilian Central Cerrado, where high temperatures and droughts are frequent [18], the choice of appropriate forage is crucial to ensure efficient soil cover, greater moisture retention, and stability in agricultural production. The biomass of Zuri and Quênia guinea grasses provided more effective and lasting soil cover than maize. This cover reduces moisture evaporation, protects against erosion, and maintains thermal stability, benefiting the development of subsequent crops such as soybean [5,31].

Higher accumulations of nitrogen, phosphorus, potassium, and sulfur were observed in the biomass of Zuri and Quênia guinea grasses at all decomposition times (Figure 3). The higher biomass production explains these results, where the accumulated nutrients were deposited in the soil and met the nutrient demands of soybean, especially nitrogen, as in its initial phase, soybean does not yet have effective biological nitrogen fixation [9]. According to Oliveira Junior et al. [32], of all the nitrogen extracted by soybean (190 to 372 kg ha^−1^), 65 to 85% comes from biological fixation, while the rest is supplied by the soil.

Forages of the *Panicum* genus, such as Zuri and Quênia guinea grasses, are known for their rapid growth and balance between the root and leaf area, where the leaves transfer photosynthates to expand the roots, optimizing water and nutrient uptake [33]. This results in higher dry mass production per hectare and nutrient accumulation.

In all cropping systems, potassium had the highest release rate, with values above 94%, and the shortest half-life (t½), as shown in Figure 3c. This phenomenon occurs because potassium is not bound to organic compounds in plant tissues and is easily released from plant residues with the first rains [34,35]. Silva et al. [7] and Silva et al. [18] also observed this high release rate and shorter half-life for potassium in different no-till soybean systems. In the present study, the rapid release of potassium from soil cover biomasses contributed to greater nutrient cycling efficiency, showing that soil cover can compensate for potassium deficiency over time and reduce the need for mineral fertilizers [36], or even eliminate it, as demonstrated by Dias et al. [19] and Muniz et al. [9].

Nitrogen and potassium are the nutrients most extracted by forages, accumulating more in the biomass, and they are also lost the most through leaching. Tropical forages, with their deep and aggressive root systems, can absorb nutrients from deep layers and release them on the soil surface, benefiting the subsequent crop [37,38].

The biomass of Zuri and Quênia guinea grasses showed the following decreasing order of nutrient accumulation: K > N > S > P. For maize biomass, the order was N > K > S > P. Cultivars of *Panicum maximum*, such as Zuri and Quênia guinea grasses, showed higher K uptake compared to N [25]. These results demonstrate that cover crop biomass aids in greater nutrient cycling, increases soil carbon [18] and nitrogen stocks [39], and can reduce production costs due to nutrient return through biomass mineralization [19].

Thus, understanding the decomposition dynamics of cover crops can provide benefits to the physical, chemical, and biological properties of the soil. This is due to the increase in carbon and organic matter, the availability of nutrients, and the reduction in soil erosion and compaction [38]. Moreover, there are improvements in the soil microbial community [40], as the diversity of plants in the cropping system positively influences organic matter quantity, litter production, and the soil rhizosphere. This contributes to greater activity of soil enzymes, which are essential to organic matter decomposition, carbon sequestration, and nutrient cycling, factors that are impacted by cropping systems [5].

For effective nutritional management in production systems, it is essential that high-quality cover crop biomass have a high nutrient concentration and that the release of these nutrients be synchronized with the growth of the subsequent crop [41]. The quantity and release rate of nutrients from plant residues are crucial, and the periods of greatest nutrient demand for plants should coincide with the peak release of biomass [9,37]. Pires et al. [42] observed that the highest nutrient accumulation in soybean plants occurs between the R5 and R7 development stages, approximately 85 days after emergence, while Carmello and Oliveira [43] indicated that most macronutrients are accumulated between 82 and 92 days, with the highest absorption rate occurring between 39 and 58 days.

In the present study, the half-life of nitrogen, phosphorus, potassium, and sulfur was 58, 48, 35, and 47; and 63, 54, 42, and 57 for the biomasses of Zuri and Quênia guinea grasses, respectively, with release rates above 75% at 120 days of decomposition, demonstrating the potential of these forages as a nutrient reserve and supply for the subsequent crop.

The highest fertilizer equivalent values were observed in the biomass of the Zuri and Quênia guinea grass cropping systems compared to maize, highlighting that these forages are more efficient in nutrient cycling, which is attributed to higher biomass production and nutrient release from plant residues.

It is worth noting that the inclusion of animals in the integrated crop–livestock system induces biotic and abiotic changes in the soil–plant–atmosphere system, impacting biogeochemical processes, especially those of carbon (C) and nitrogen (N) [44]. This system promotes synergy between soil, plants, and animals, improving biomass production, productive efficiency, and soil fertility through nutrient cycling [20]. The excretion of manure and urine by animals contributes to nutrient return to the soil, stimulating forage regrowth and increasing organic matter [19,45]. Cherubin et al. [46] also reported that carbon cycling in pasture areas, intensified by the decomposition of deep grass roots and the incorporation of organic material, is crucial for soil carbon sequestration, especially in well-managed systems, where C stabilization can reach levels comparable to those observed in native vegetation.

The use of these forages as cover crops not only improves nutrient availability for the subsequent crop but can also reduce the need for mineral fertilizers, resulting in cost savings and lower environmental impact [9]. The adoption of cover crops like Zuri and Quênia guinea grasses in no-till systems in the tropics can be an effective strategy to increase nutrient use efficiency and reduce production costs while contributing to agricultural sustainability.

The agronomic characteristics and soybean yield were not influenced by the different cropping systems, showing similar results between the biomass of Zuri and Quênia guinea grasses and maize. The lack of significance among the systems can be explained by uniform precipitation during soybean development, without periods of water deficit. During this period, regular rains were observed, and combined with good soil fertility and proper cultural practices, the forage biomass systems did not affect productivity. Previous studies conducted in the same area by Dias et al. [19] and Muniz et al. [9] found that in ICLS systems, there was an increase in soybean production compared to the soybean–maize succession system in more challenging years with drought periods.

However, the Zuri and Quênia guinea grass cropping systems were more effective for nutrient cycling compared to maize, with some of the nutrients being returned to the soil through the mineralization process (Figure 4), which is essential for the sustainability of no-till systems. In addition, tropical forage cover plays an important role in weed suppression and improving conditions for successive crops [47], resulting in cost savings and lower environmental impact [20].

Through correlation and PCA analysis, it was possible to better understand the response pattern of the variables. The positive correlation among the variables in group 1 (biomass, nitrogen, phosphorus, potassium, sulfur, equivalent N, equivalent P_2_O_5_, and equivalent K_2_O) indicates that an increase in one variable directly affects the others in the same direction. Positive and high correlations among these variables have already been observed by Silva et al. [20] when evaluating the efficiency of nutrient release in the biomass of *Panicum* cultivars in integrated systems for soybean productivity.

The negative correlation of the C:N ratio with the variables in group 1 indicates that they have an inverse relationship, i.e., an increase in the C:N ratio causes a reduction in these variables. Negative and high correlations among these variables have already been observed by Silva et al. [7], where a high C:N ratio results in lower nutrient availability due to immobilization during the decomposition process.

The results showed that productivity had little correlation with the other variables. Despite the advantages in terms of nutrient cycling and soil structuring promoted by the forages, final soybean productivity did not differ statistically among the treatments. This result can be attributed to soil fertility saturation, which limited the potential for yield increase [48]. Furthermore, another influencing factor is that there was no significant climatic challenge during the experiment. If there had been, the integrated crop–livestock system could have provided higher soybean productivity, as integrated systems maintain greater soil moisture, offering greater resilience to climatic stresses [49] and mitigating the effects of extreme climatic events [50], since a water deficit is the main cause of soybean yield losses in Brazil [51,52].

Thus, it is suggested that integrated crop–livestock systems with Zuri and Quênia guinea grasses are viable alternatives to increasing the sustainability and resilience of the cropping system. These findings reinforce the importance of selecting appropriate cover crops for no-till and integrated crop–livestock systems, aiming not only to maximize productivity but also to promote long-term sustainability of agricultural systems, encompassing regenerative livestock practices.

## 4. Materials and Methods

### 4.1. Area Description

The experiment was conducted at the Centro Tecnológico Comigo (CTC), in Rio Verde, Goiás, Brazil, from February 2023 to March 2024, covering two agricultural seasons under the coordinates 17°45′48″ S and 51°02′14″ W at an altitude of 832 m. The soil was classified as a typical Dystrophic Red Latosol [53], with 383.4, 71.25, and 354.65 g kg^−1^ of clay, silt, and sand, respectively.

During the experiment, precipitation and average monthly temperature data were monitored (Figure 7). An average temperature of 22.9 °C and precipitation of 1882.7 mm were observed, with regular rainfall distribution.

### 4.2. Experimental Design, Treatments, and Crop Establishment

The experimental design used was a randomized complete block design with four replications. Three treatments were evaluated: The first and second consisted of biomass production of Zuri guinea grass (*Panicum maximum* cv. BRS Zuri) and Quênia guinea grass (*Panicum maximum* cv. BRS Quênia) in integrated crop–livestock systems; the third treatment was maize cultivation in succession to soybean.

**Figure 8:**
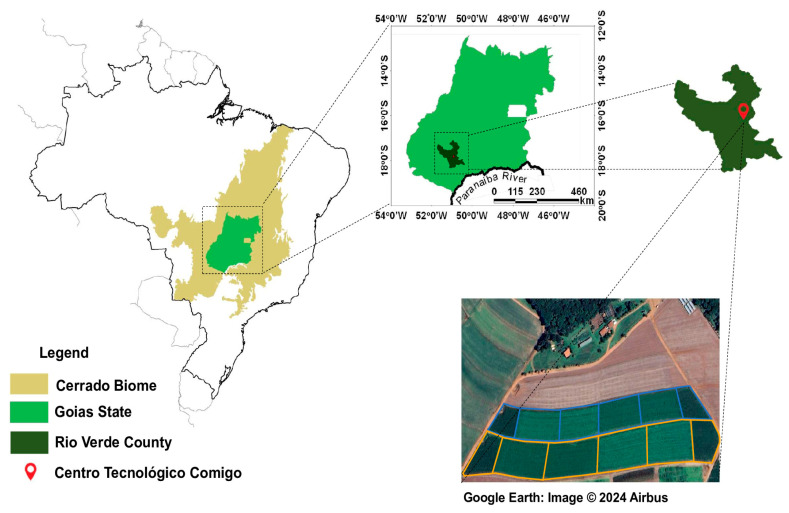
Aerial view of the experimental area (source: Google Earth). The orange lines delineate block 1, and the blue lines delineate block 2.

The total area used was 5.14 ha, with 2.93 ha (block 1) and 2.21 ha (block 2). The field corresponding to block 2 has been using the integrated crop–livestock system since the 2011/2012 agricultural year, and the field corresponding to block 1 since the 2016/2017 agricultural year (Figure 8). The experimental plots consisted of twelve observation units divided into two blocks, with four plots for each forage evaluated and four plots with maize. The size of each experimental plot was 488.3 m^2^ and 336.8 m^2^ for block 1 and block 2, respectively.

**Figure 9:**
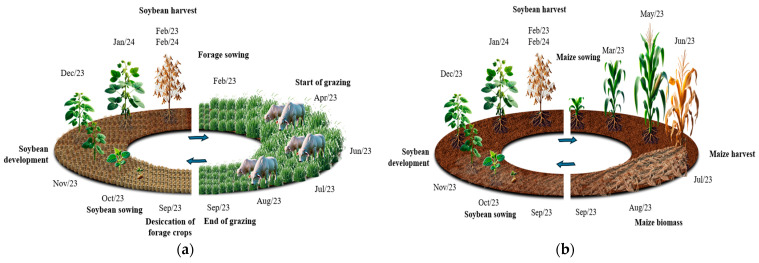
Schematic representation of the cropping systems with *Panicum* genus forages in an integrated crop–livestock system (**a**) and the maize cropping system in succession to soybean (**b**).

Figure 9 illustrates the cropping systems with *Panicum maximum* genus forages (Zuri and Quênia guinea grasses) in an integrated crop–livestock system (Figure 9a) and the maize cropping system in succession to soybean (Figure 9b), with activity diversification, maximizing land use throughout the year.

The first phase of the research began after the harvest of the 2022/2023 soybean crop. After harvesting, the *Panicum* cultivars (BRS Zuri and BRS Quênia) were sown for subsequent grazing by the animals. Simultaneously with the sowing of the forages, maize was sown as a second crop. Maize was used as a reference to indicate productivity and viability in relation to the forage and animal production areas practiced at the same time.

The forages were sown on 26 February 2023, using a Baldan SPDE CXP 5000 pneumatic seeder–fertilizer with 24 rows and 17 cm row spacing, at a depth of 3 cm. A seed rate of 8.8 kg ha^−1^ was used, with a cultural value of 80% for Zuri and Quênia guinea grasses. For sowing fertilization, a nitrogen, phosphorus, and potassium (NPK) fertilizer (11-40-00) was applied at a rate of 420 kg ha^−1^.

Maize (hybrid B2401 PWU) was sown on 27 February 2023, at a rate of 2.8 seeds m^−1^, using a Vence Tudo Panther SM 7000 pneumatic seeder–fertilizer with 6 rows and 50 cm row spacing, mounted on a tractor (6155 J, 115 hp, John Deere). During sowing, 150 mL ha^−1^ of the co-inoculant Biomax Azum (*Azospirillum brasilense*, minimum concentration 3.0 × 10^3^ CFU m^−1^, Vittia) and 500 mL ha^−1^ of Meta Turbo (*Metarhizium anisopliae*) were applied in the planting furrow. Sowing fertilization was performed with 400 kg ha^−1^ of NPK 15-15-15 fertilizer. On 16 March 2023, a top-dressing fertilization with 150 kg ha^−1^ of nitrogen from urea was applied. The maize was harvested on 10 July 2023, with a development cycle of 133 days.

On 28 April 2023, 61 days after the forage sowing, 16 male Nellore cattle with an average weight of 251 ± 17.15 kg were introduced into the system for grazing. The animals were weighed after a 16 h fast from solid food, then randomly assigned and distributed among the treatments. Stocking rates were variable and adjusted as needed throughout the experiment, according to forage availability. Initial and final stocking rates were 2.95 and 3.14 animal units ha^−1^ for Quênia guinea grass, and 3.35 and 3.19 animal units ha^−1^ for Zuri guinea grass. The animals remained in the system for 145 days, until 20 September 2023 (off-season period), in an intermittent grazing system, with a 7-day grazing period and a 21-day rest period for the forage, completing five grazing cycles. After the animals were removed from the area, the forages regrew, and subsequently, desiccation was performed to generate biomass for soil cover.

### 4.3. Desiccation Efficiency, Biomass Production, Decomposition, and Nutrient Accumulation

The desiccation of the forage grasses was carried out on 22 September 2023, using 2.5 L ha^−1^ of Roundup Ultra^®^ (Glyphosate–Ammonium Salt, 715 g L^−1^) in the ICLS areas. Herbicide efficiency was evaluated based on the criteria established by the Brazilian Society of Weed Science—SBCPD, according to Gazziero [54]. Control assessments were conducted at 7, 14, and 21 days after herbicide application, using a visual scale from 0 to 100%, where 0% corresponds to no injury and 100% to plant death.

To quantify biomass production, eight samples of mulch were collected one day before soybean sowing, using a 1.0 × 1.0 m (1 m^2^) square randomly distributed within each plot. The material was cut at ground level, then weighed, and the samples were placed in a forced-air oven at 55 °C until constant weight was reached, with the quantities extrapolated to kg ha^−1^.

Biomass decomposition was evaluated using nylon “litter bags” with a 2 mm mesh and dimensions of 25 × 30 cm [55]. Four bags containing biomass of each species in proportion to the dry biomass produced per hectare were placed in direct contact with the soil. At 30, 60, 90, and 120 days after the management of decomposition, one “litter bag” was removed from each plot to assess the remaining biomass and determine biomass decomposition over the 120-day period. At each evaluation, the material was sent to the laboratory for the removal of adhered soil using running water until all residues were removed, then dried in an oven at 55 °C until a constant weight was achieved to obtain the dry biomass. Based on the initial biomass production data (kg ha^−1^) of the systems, decomposition percentages were calculated as the ratio between the mass of the litter bags in kg ha^−1^ and the initial biomass production [19].

The biomass samples were initially dried in an oven at 55 °C until a constant weight was reached and then ground in a mill with 1 mm blades for homogenization. For nutrient determination, we used the nitric–perchloric digestion method described by Malavolta et al. [56]. In this procedure, approximately 0.5 g of the dried and ground sample was added to a digestion tube containing 4 mL of concentrated nitric acid (65%) and heated to 95 °C in a digestion block, promoting initial digestion. Then, 2 mL of perchloric acid (70%) were added, and the temperature increased to 150 °C until the extract became clear and colorless, indicating complete digestion. The extract was then diluted to a standard volume (25 mL) with distilled water for further analysis. Specific methods were used for nutrient quantification: Phosphorus (P) was quantified by spectrophotometry using ammonium molybdate and ascorbic acid, forming a blue complex measurable by absorbance at 765 nm; potassium (K) was determined by flame photometry, utilizing the characteristic light emission of potassium when excited at high temperatures; sulfur (S) was quantified by turbidimetry, with the formation of insoluble complexes allowing turbidity quantification by spectrophotometer absorbance at 420 nm; nitrogen (N) was determined by sulfuric digestion; and carbon (C) was indirectly quantified by mass loss during incineration in a muffle furnace at 550 °C for 4 h. Subsequently, the carbon/nitrogen (C:N) ratio of the material was calculated. To evaluate nutrient accumulation in the biomass, macronutrient concentrations were multiplied by biomass production, expressing the results in kg ha^−1^.

The fertilizer equivalents of N, P_2_O_5_, and K_2_O in the soil cover biomass of the cropping systems were determined considering the atomic mass of the elements according to analytical chemistry conventions and the N, P, and K concentrations of the analyzed residues [19].

### 4.4. Soybean Establishment in the 2023/2024 Season and Crop Management

For the establishment of the 2023/2024 soybean crop, soil samples were collected from the 0–20 cm layer, and their chemical properties were as follows: pH in CaCl_2_ (calcium chloride): 5.1; Ca (calcium): 2.8 cmol_c_ dm^−3^; Mg (magnesium): 0.76 cmol_c_ dm^−3^; Al (aluminum): 0.08 cmol_c_ dm^−3^; Al+H (aluminum + hydrogen): 3.3 cmol_c_ dm^−3^; K (potassium): 0.39 cmol_c_ dm^−3^; CEC (cation exchange capacity): 7.3 cmol_c_ dm^−3^; V1: 53.91%; P (phosphorus) (Mehlich): 40.57 mg dm^−3^; S (sulfur): 6.2 mg dm^−3^; Cu (copper): 1.63 mg dm^−3^; Zn (zinc): 1.6 mg dm^−3^; Fe (iron): 24.8 mg dm^−3^; OM (organic matter): 25.65 g kg^−1^.

Before sowing, 1500 kg ha^−1^ of filler limestone (100% TRNP) were broadcast over the entire experimental area on 27 September 2023, and 1585 kg ha^−1^ of gypsum on 28 September 2023. The soybean was sown mechanically in the different cropping systems on 17 October 2023. The variety used was CZ37B43 IPRO, with a row spacing of 0.50 m and a sowing rate of 15 seeds m^−1^. During sowing, 120 kg ha^−1^ of NPK 05-25-25 fertilizer were applied in the planting furrow. On 9 November 2023, 76 kg ha^−1^ of KCL (potassium chloride) were broadcast in all treatments. When the plants were at the V3 and V4 development stages, a foliar application of 300 mL ha^−1^ of a product containing Co (cobalt): 0.5%, Mo (molybdenum): 2.5%, Zn (zinc): 1.5%, and Fe (iron): 0.5% was performed.

Fungicide applications were carried out 40 days after sowing (DAS) (dose of 0.3 L ha^−1^ of pyraclostrobin and 0.5 L ha^−1^ of mineral oil) and at 60 DAS (dose of 0.2 kg ha^−1^ of Elatus (60 g of azoxystrobin and 30 g of benzovindiflupyr) and 0.6 L ha^−1^ of mineral oil).

### 4.5. Statistical Analysis

The results for desiccation efficiency were fitted using regression equations, with standard error. To describe biomass decomposition and nutrient accumulation, the data were fitted with standard error to an exponential mathematical model (y = aekx) and a linear model for the C:N ratio (y = a + bx) using SigmaPlot software version 10. Comparisons between the estimated equations were performed according to the procedure described by Snedecor and Cochran [57], which tests data homogeneity (F) and the significance of the angular (0.4343k) and linear coefficients (log a) of the linearized equations (log y = log a + 0.4343kx).

To calculate the half-life (t₁/₂), that is, the time required to decompose 50% of the remaining biomass, the equation proposed by Paul and Clark [58] was used: t₁/₂ = 0.693/ k, where t₁/₂ is the half-life of the dry biomass and k is the dry biomass decomposition constant.

The results for nutrient concentration, fertilizer equivalent, soybean agronomic characteristics, and grain yield were subjected to analysis of variance using R software version R-3.1.1 (2014), employing the ExpDes package [59]. Means were compared using Tukey’s test at a significance level of 5% probability. Principal component analysis (PCA) was performed using the “tidyverse”, “stats”, and “factoextra” packages in R software version R-3.1.1 (2014).

## 5. Conclusions

The results indicate that Quênia guinea grass has higher desiccation efficiency. Both forages (Zuri and Quênia guinea grasses) can be recommended for integrated crop–livestock systems, as they show similar biomass production and nutrient accumulation.

Soybean productivity was not influenced by the different cropping systems, showing similar results between the biomass of Zuri and Quênia guinea grasses and maize. However, forage biomass enriches the soil through the return of fertilizer equivalents, which can be considered in future studies for reducing mineral fertilizers and ensuring greater sustainability of agricultural systems.

## Figures and Tables

**Table 1:** Plant height, height of the first pod insertion, number of pods per plant, number of grains per pod, 1000-grain weight, and soybean yield under the biomass of Zuri and Quênia guinea grasses and maize.

Cultivation System	Plant Height (cm)	Insertion 1st Pod (cm)	Pod/Plant Number
Zuri guinea grass	94.57	13.35	33.07
Quênia guinea grass	92.25	12.70	34.11
Maize	89.67	11.37	33.40
SEM	0.534	0.559	0.501
*P-value*	0.109	0.111	0.118
	**Number of grains/pods**	**1000-grain weight** **(g)**	**Soybean Yield (kg ha^−1^)**
Zuri guinea grass	2.50	204.7	4966
Quênia guinea grass	2.63	202.4	5040
Maize	2.60	198.2	5106
SEM	0.033	3.102	69.15
*P-value*	0.161	0.169	0.065

SEM: mean standard error.

**Table 2:** Correlation between agronomic variables and nutrients in the principal component analysis (PCA) and contributions of the principal components.

Variables	CP1	CP2
Biomass	0.99	−0.07
CN	−0.95	−0.02
Nitrogen	0.99	0
Phosphorous	0.99	−0.1
Potassium	0.99	−0.06
Sulfur	0.99	0.01
Equivalent N	0.99	0
Equivalent P_2_O_5_	0.99	−0.1
Equivalent K_2_O	0.99	−0.06
Productivity	−0.41	−0.91
Eigenvalue	8.95	0.86
Variance	89.53	8.62
Cumulative variance	89.53	98.15

Parameters: phosphorous: phosphorus concentration, biomass: biomass accumulation, potassium: potassium concentration, equivalent K_2_O: equivalent concentration of K_2_O, nitrogen: nitrogen concentration, equivalent N: equivalent concentration of nitrogen, sulfur: sulfur concentration, CN: carbon/nitrogen ratio, productivity: crop yield.

## Data Availability

The original contributions presented in the study are included in the article; further inquiries can be directed to the corresponding author(s).
